# Effect of thermal osteonecrosis around implants in the rat tibia: numerical and histomorphometric results in context of implant removal

**DOI:** 10.1038/s41598-022-25581-9

**Published:** 2022-12-23

**Authors:** Kristian Kniha, Benita Hermanns-Sachweh, Faruk Al-Sibai, Reinhold Kneer, Stephan Christian Möhlhenrich, Marius Heitzer, Frank Hölzle, Ali Modabber

**Affiliations:** 1 Department of Oral and Cranio-Maxillofacial Surgery, University Hospital, RWTH Aachen, Pauwelstraße 30, 52074 Aachen, Germany; 2 Private Clinic for Oral Surgery Dres. Kniha, Rosental 6, 80331 Munich, Germany; 3 Private Institute for Implant Pathology, ZBMT, Campus Melaten, Pauwelsstaße 17, Aachen, Germany; 4 Institute of Heat and Mass Transfer, RWTH Aachen University, Augustinerbach 6, Aachen, Germany; 5 Department of Orthodontics, University of Witten/Herdecke, Alfred-Herrhausen Str. 45, 58455 Witten, Germany

**Keywords:** Cell biology, Cell death, Apoptosis, Bone development, Bone remodelling

## Abstract

The purpose of this rat study was to explore the feasibility of in vivo temperature thresholds affecting bone contact at the implant surface. Based on these data, thermal necrosis should be used for implant removal in the subsequent in vivo study. Rat tibiae of 48 animals at one site were randomly treated with heat or cold before implant insertion. Temperatures of 4 °C, 3 °C, 2 °C, 48 °C, 49 °C and 50 °C for a tempering time of 1 min were evaluated. Numerical simulations of the heat source-implant-bone system were carried out. Effects were assessed by histomorphometrical measurements. The results showed that the selected method of direct tempering using a tempering pin was suitable for maintaining a uniform layer around the pin. Starting at warm temperatures of 48 °C and rising to 50 °C, the BIC ratio revealed declining values and a significant difference was observed when comparing 50 °C to the control group (p = 0.03). However, there were no significant variations within the cold temperatures. This study pinpointed temperature discovered that could lead to the thermo-explantation and so that the number of samples used in future studies on temperature-induced bone necrosis can be reduced to a minimum. Significant BIC value reduction was seen at a temperature of 50 °C for 1 min.

## Introduction

Dental screw-type implants osseointegrate into the jawbone and thus achieve the necessary strength to be able to support dental prosthesis constructions^1^. Due to numerous long-term studies as an implant material, titanium is the current gold standard^2–5^. Titanium proved to be a biologically suitable material on which chemical compounds can connect with the surrounding tissues, which are also sufficiently biomechanically stable^1^.

Periodontitis is a risk indicator for peri-implant diseases. Patients can be classified by Stage (I, II, III, IV) and Grade (A, B, C)^6,7^. Furthermore, Rosen et al. suggested a modified classification, including information regarding the implant position, as emerging information suggests that this is a crucial factor in the etiology and prognosis of peri-implantitis^8^. Based on that prognosis of peri-implantitis implants may have to be removed. The methods for implant removal consider first the atraumatic removal of the implants by applying counter torque with the implant driver, and if not feasible, applying methods based in osteotomy like drilling around the implant, trephine, or induced thermal necrosis^9–11^.

This can lead to an additional bone defect around the implant bed. This defect might considerably worsen the possibility of a new implant restoration. As a more bone-preserving procedure, special instruments, such as individual ratchets for unscrewing, have been developed. However, the connection between bone and implant must be broken mechanically. The applied force is usually 10–15 times higher than when screwing in the implants. Depending on the implant geometry, surface texture and bone quality, among other factors, torque out resistances of more than 300 Ncm may occur around fully osseointegrated implants, especially in cases with wrong implant positioning^12^.

Erikssen and Albrektsson have presented threshold value of 47 °C and 1 min that will induce bone necrosis^13^. Several case reports showed that implants were easily removed after an initial random thermal treatment resulting in minimal osteonecrosis of the jaw^14–16^. Weather this procedure is safe and predictable is not clear yet. The purpose of this pre-clinical in vivo study in rat tibia was to lay a foundation for a future in vivo study in pigs to focus on the potential of thermally induced osteonecrosis for dental implant removal. This in vivo rat study is based on the results of a previous cadaveric pilot study that evaluated a feasible temperature threshold that affects the osteocyte cells and the jawbone matrix in the upper and lower jaws using both warm and cold temperatures^17^. The cadaveric results indicated that temperature/time levels of 51 °C for 10 s for warm temperatures and 5 °C for 30 s for cold stimuli showed significant matrix degeneration and that these levels may be used for thermo-explantation. This rat study was conducted to verify these results in an in vivo setting with non-integrated implants and to reduce these sample sizes for the subsequent in vivo pig study with realistic osseointegrated implants.

Therefore, the primary aim of this study was to analyze the effects of feasible cold and worm temperature/time intervals on the rat tibia. The authors hypothesized that an appropriate temperature/time interval for warm and cold temperatures leads to a reduced bone-to-implant contact (BIC) ratio around implants.

## Methods

**Figure 1:**
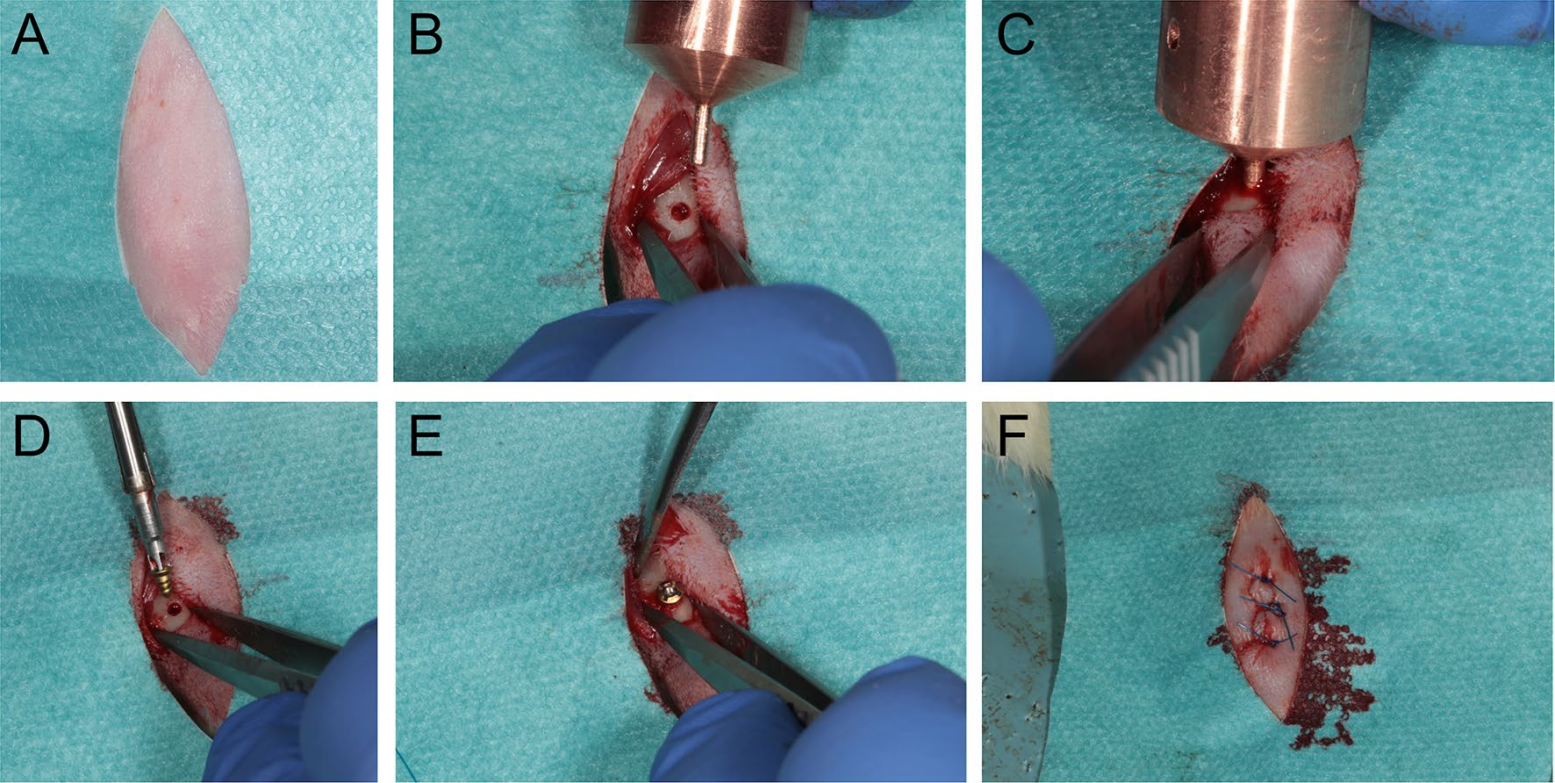
(**A**) Before surgery the skin was shaved, disinfected, and cut with a scalpel after sterile draping. Implantation was performed after exposure of the tibia by predrilling with a pilot drill with a 1.5 mm diameter under strict cooling with sterile saline (Medartis AG, Basel, Switzerland). In the case of the test group, the thermal treatment took place after predrilling and before implant placement. The device exhibited a congruent clamp fit in the drill studs. After a primary stable insertion of the implants, the wounds were closed with suture material and a follow-up of 7 days was performed. (**B**, **C**) Implantation was performed after exposure of the tibia by predrilling with a pilot drill. In the case of the test group, the thermal treatment took place after predrilling and before implant placement. (**D**, **E**) Primary stable insertion of the implants was achieved in all cases. (**F**) The wounds were closed with suture material and a follow-up of 7 days was performed.

During the preliminary numerical investigation, the ideal tempering method in the rat tibia was studied. In this pilot study, two tempering pistons capable of generating warm or cold temperatures with integrated thermocouples were developed at the Institute of Heat and Mass Transfer of the University. Warm and cold temperatures were generated with a device (circulating bath thermostat) incorporating in the water circuit (Fig. 1). In the run-up to the experimental investigation, the influence of tempering was investigated in advance with the help of numerical simulations of the heat source-implant-bone system. The simulations were carried out with COMSOL (COMSOL Multiphysics GmbH, software COMSOL Multiphysics, Göttingen^18^). COMSOL uses the finite element method to model physics-based processes. The "heat transfer module" used to calculate heat propagation in the rat bone allows the modeling of heat transport in the form of heat conduction, convection, and thermal radiation^18^.

**Figure 2:**
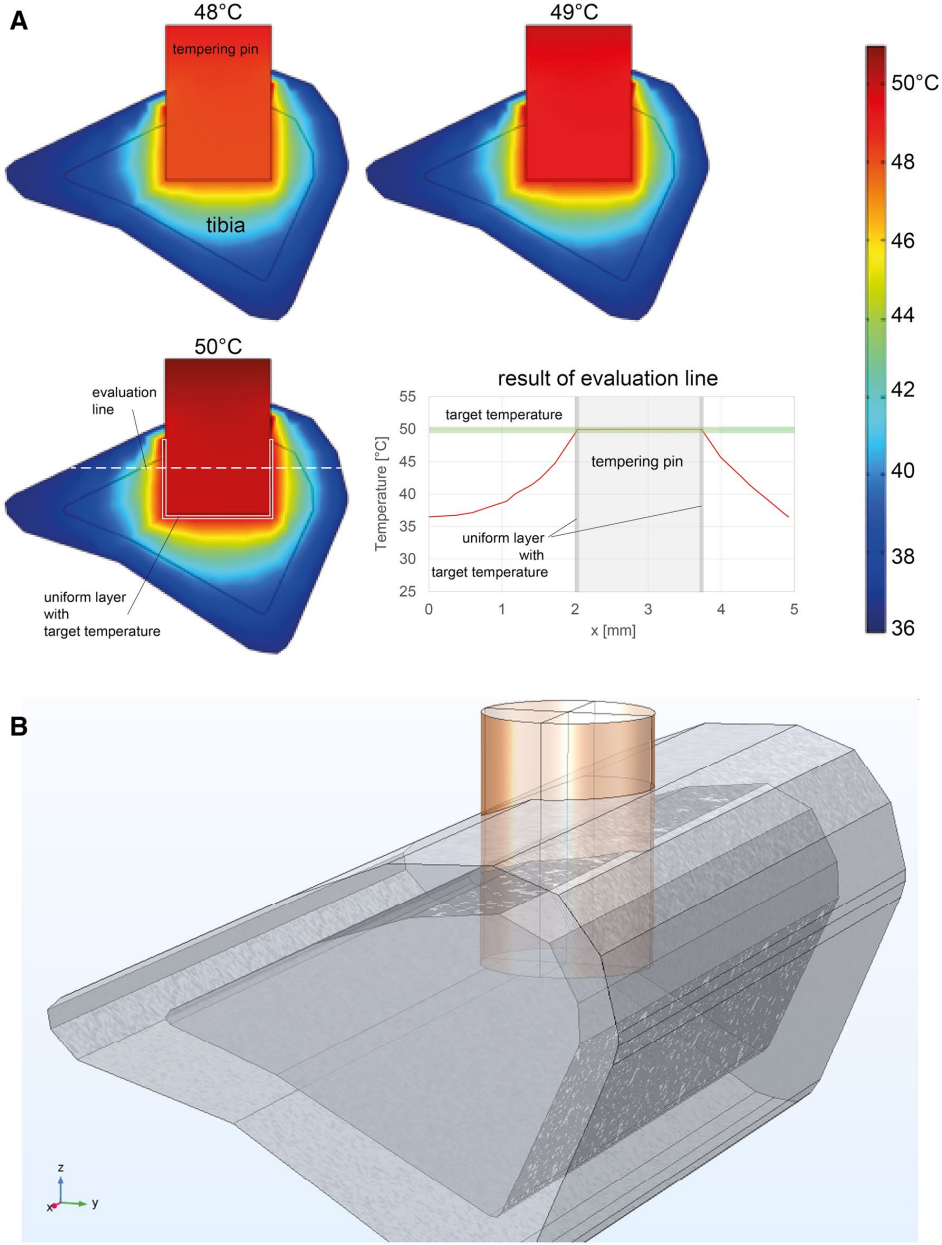
(**A**) 3D model of the tempering pin in rat bone. (**B**) 3D simulations of the tempering pin in rat bone. Exemplarily the warm temperatures are represented here.

The following initial and boundary conditions were selected for the simulation: The starting temperature for the bone and implant was 37 °C, for the skin 33 °C, while the starting temperature of the adapter was adjusted for each simulation run. The heat losses of the skin, the adapter and the implant to the environment were modeled as convective heat flows (Fig. 2A, Table 1). The investigation was carried out to compare a direct tempering of the mini-screw with an indirect tempering pin before implantation.

**Table 1:** Table with thermophysical material properties and simulation boundary conditions.

Tempering pin	c = 1688.1 J/(kg K) r = 1648.1 kg/m^3^ l = 0.401 W/(m K)
Spongiosa	c = 1688.1 J/(kg K) r = 1648.1 kg/m^3^ l = 0.401 W/(m K)
Compacta	c = 1313 J/(kg K) r = 1908 kg/m^3^ l = 0.32 W/(m K)
Boundary conditions	Thermal resistance: R = 6.0E−6 (m^2^ K)/W Natural convection on pin surface: T = 20 °C Start temperature of bone: T = 36.5 °C Bone outer temperature (fix): T = 36.5 °C

### Experimental protocol

At the beginning of the study, 48 adult male Sprague–Dawley rats, each weighing 450 g and aged four months (Janvier Labs, Le Genest-Saint-Isle, France), were included. One examiner performed the individual experimental steps of the study. This investigation was carried out in accordance with the guidelines of the European Parliament and of the Council on the protection of animals used for scientific purposes, ARRIVE (Animal Research: Reporting of In Vivo Experiments)^19^, and Directive 2010/63/EU. We confirm that the experimental protocol was approved by a named institutional and licensing committee (name of the appropriate local authority Landesamt für Natur und Verbraucherschutz, Recklinghausen, Germany; Ref. 2019A276).

In each group, six temperatures with eight animals were randomly tested. Each animal received one test implant in one tibia whereas the contralateral side was used for a control implant (Medartis AG, Modus 2.0 3 mm cortical screws, Basel, Switzerland). Therefore, for each temperature, eight tests and eight control samples were investigated, resulting in a total of 12 groups with 96 implants. All procedures were performed under general anesthesia. Thirty minutes before the start of surgery, the weight of the animals was determined. Then the animals received 0.03 mg subcutaneous buprenorphine (0.1 ml Temgesic/kg body weight, remedix GmbH, Germany). Induction of anesthesia was done by the inhalation of narcotic in an induction box with an isoflurane (4 vol%)-oxygen (vol. 30%) air mixture (Isofluran, 2.5–5 vol% Piramal GmbH, Hallbergmoos, Germany). The continuation of inhalation narcosis with an isoflurane (2 vol%) oxygen (30%) air mixture was performed via a nasal mask. After the induction of anesthesia, the animals were positioned supine on an adjustable warming mat.

One tibia of each animal was randomly tempered as a test side before insertion. The contralateral side served as a control group without tempering. The six test groups were divided into cold and warm temperatures, including 4 °C, 3 °C, 2 °C, 48 °C, 49 °C and 50 °C, for a tempering time of 1 min. The division was based on the results of a previous study^20^. Before the temperature application and implantation, the skin was shaved, disinfected, and cut with a scalpel after sterile draping (Fig. 1). Implantation was performed after exposure of the tibia by predrilling with a pilot drill with a 1.5 mm diameter under strict cooling with sterile saline (Medartis AG, Basel, Switzerland). In the case of the test group, the thermal treatment took place after predrilling and before implant placement. The device exhibited a congruent clamp fit in the drill studs. After a primary stable insertion of the implants, the wounds were closed with suture material and a follow-up of 7 day was performed. Every day postoperatively, the animals were treated once a day with carprofen 4 mg/kg subcutaneously (Rimadyl, Zoetis GmbH, Berlin, Germany), according to a score sheet. The animals were sacrificed at the end of the study under deep isoflurane anesthesia by cervical dislocation.

### Histomorphometric analyses

The animals were finalized after 7 days by cervical dislocation under deep isoflurane anesthesia (4%). A follow-up time of 7 days was selected to first determine the thermal effect on the bone in the living tissue around implants^21^. The tibia samples were stored in 4% formalin (neutrally buffered with methanol) for 48 h (Otto Fischar GmbH & Co. KG, Saarbrücken, Germany). The samples were dehydrated using ascending ethanol gradients (50–100%) prior to embedding them in methylmethacrylate resin (Technovit 9100, Heraeus Kulzer GmbH, Frankfurt, Germany). Coronal sections of the embedded undecalcified specimens were obtained at a thickness of about 200 µm using an EXAKT cutting unit (EXAKT Technologies Inc., Oklahoma City, Oklahoma, USA). It was then thinned and polished manually to a final thickness of about 50–70 µm^22^. Final specimens were stained with toluidine blue according to the protocol and were analyzed using light microscopy. One slide for each implant was obtained in the coronal section through the implant center.

The tissue structures were analyzed by one specialized pathologist using digital microscopy. The set of parameters evaluated in the histomorphometric analysis included a quantitative evaluation of the periimplant bone. With the qualitative evaluation, exact measured values could be recorded. In contrast, a score system was used in the semi quantitative measurement method. The area to be examined was the directly surrounding peri-implant hard tissue. The evaluation method was based on a previously published method^20^. The bone-to-implant ratio was calculated by measuring (μm) the complete circumference in the sectioning of the implant and then recording the area with a histologic bone contact. The bone necrosis was evaluated based on empty osteocyte lacunae. In the case of bone necrosis, the layer thickness at the maximum distance at a 90° angle to the implant surface was measured quantitatively in μm. Additionally, semiquantitative analyses were performed for several parameters. On the one hand connective tissue proliferation and on the other hand formation of new bone were investigated (0 = normal, 1 = minimal count, 2 = progressing count and 3 = severe amount). All parameters were examined under 40 × to 600 × magnification with the OLYMPUS digital microscope DSX-1000 and the integrated morphometric stream desktop software (Olympus Hamburg, Germany)^23^.

### Statistical analysis

Analyses were performed using Prism 8 software for Mac OS X (GraphPad; La Jolla, CA, USA) running on Apple OS X. Variables were analyzed using the Kolmogorov–Smirnov normality test. Kruskal–Wallis and Dunn’s multiple comparison tests with adjustments were used to identify differences between parameters.

Post hoc power analysis was performed with the G-Power software (G-Power, Heinrich-Heine-Universität, Düsseldorf, Germany) using the post hoc ANOVA test by means of groups to determine the power of 100% (primary study aim BIC) based on the total sample size of 48 animals using an effect size of 5.3 and an α of 0.05.

### Ethical approval

The study protocol received ethical approval from the appropriate local authority (Landesamt für Natur und Verbraucherschutz, Recklinghausen, Germany; Ref. 2019A276).

### Informed consent

For this type of study, formal consent is not required.

## Results

The first numerical attempts to directly temper the mini-screw in the rat tibia were discarded due to physical limitations, as too much temperature loss was evaluated, especially at cold temperatures. The antifreeze liquid used was by far not sufficient for this miniature approach. Figure 2B shows the results of the 3D simulations of the tempering pin in the rat bone as a 2D temperature distribution in a sectional plane across the femur bone and the rectangular tempering pin. Different heating (48 °C, 49 °C and 50 °C) and cooling (1 °C, 2 °C and 3 °C) scenarios were varied. Furthermore, results of the numerical investigation show that the selected method of direct tempering by means of a tempering pin is suitable for maintaining a uniform layer around the pin at the target temperature in a time interval of 60 s.

**Figure 3:**
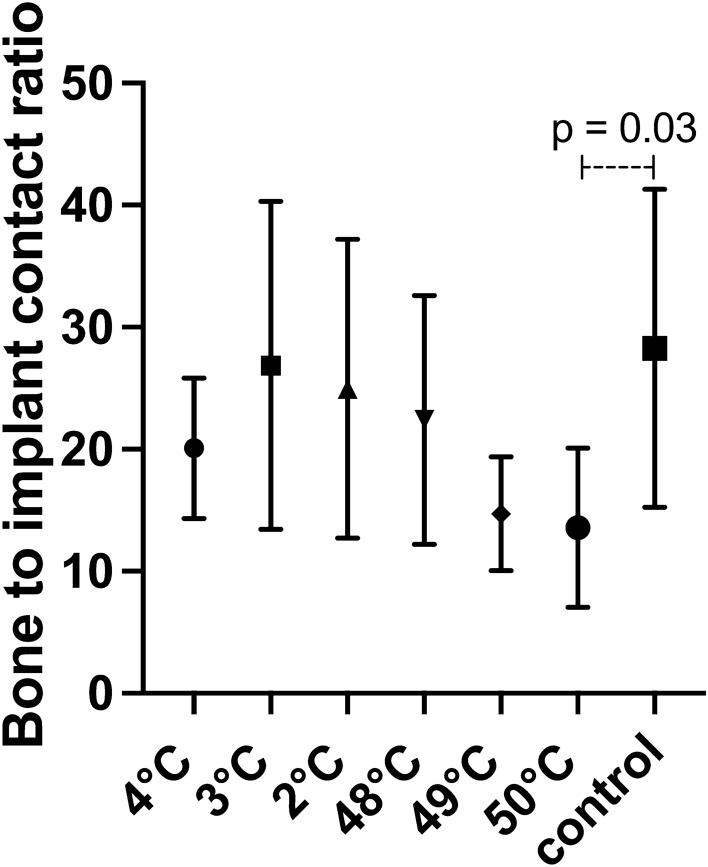
The bone-to-implant ratio (BIC) was calculated by measuring (μm) the complete circumference in the sectioning of the implant and then recording the area with histologic bone contact.

The BIC ratio indicated decreasing values starting at warm temperatures of 48 °C and rising to 50 °C (Fig. 3 and Table 2); a significant difference was noted when comparing 50 °C to the control group (p = 0.03). However, no significant differences were noted regarding cold temperatures.

**Table 2:** This table showed the descriptive data (mean and standard deviation) of the BIC ratio, depth of necrosis, connective tissue proliferation and new bone formation around the implant bodies.

	Temperature input for 1 min						
	4 °C	3 °C	2 °C	48 °C	49 °C	50 °C	Control
**BIC %**							
Mean	20.10	26.86	24.96	22.41	14.71	13.57	28.28
SD	5.75	13.44	12.23	10.19	4.66	6.52	13.02
**Distance of necrosis (μm)**							
Mean	225.00	267.10	211,60	305.10	331.80	351.60	184.70
SD	62.04	54.46	111.60	113.10	245.90	203.10	125.20
**Connective tissue proliferation (score)**							
Mean	1.47	1.50	1.58	1.34	1.21	1.96	1.19
SD	0.74	0.53	0.74	0.58	0.27	0.60	0.69
**New bone formation (score)**							
Mean	2.11	2.22	1.00	1.78	2.04	1.79	1.87
SD	0.53	0.39	0.76	0.51	0.85	0.81	0.63

**Figure 4:**
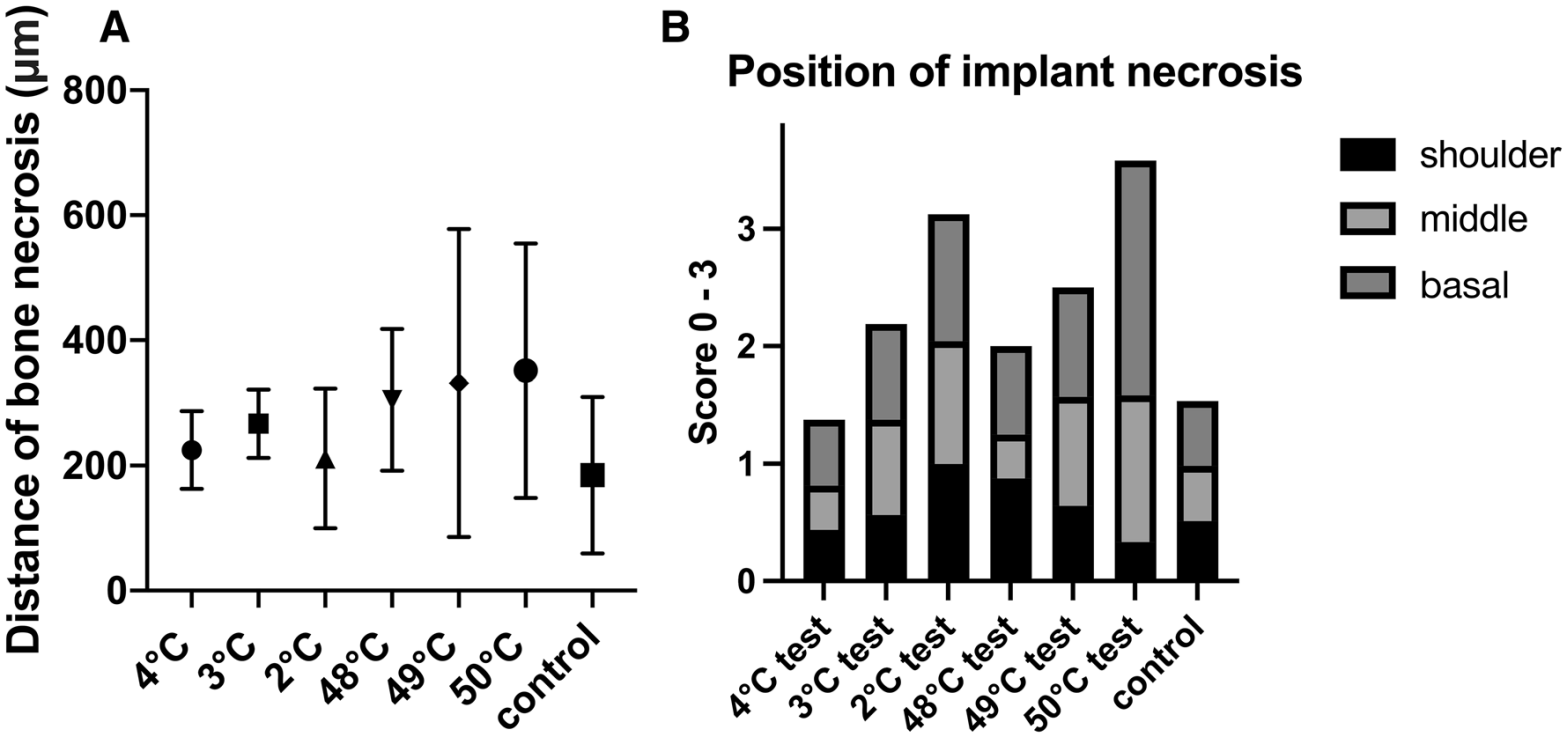
(**A**) Bone necrosis was measured at the maximum distance in a 90° angle to the implant surface in μm. (**B**) Additionally, the frequency distribution of necrosis sites at the implant shoulder, middle or tip were evaluated.

The distance of bone necrosis around the implant revealed increasing values with rising temperature damage, for example, decreasing cold temperatures and increasing warm temperatures, albeit with no statistical significance (Fig. 4A).

The site of bone necrosis around the implant was distributed relatively evenly at cold temperatures to the shoulder area, the middle and the implant tip (Fig. 4B). By contrast, 50 °C showed increased necrosis on the tip and hardly any on the shoulder.

**Figure 5:**
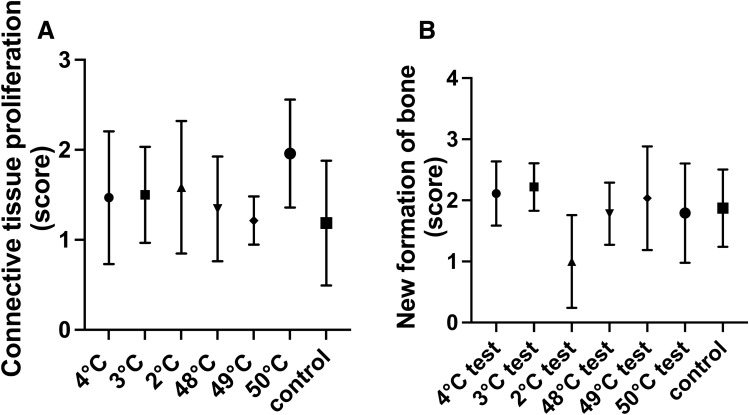
(**A**) The proliferation of connective tissue was evaluated with a score (0 = to 3 = severe amount). (**B**) The new formation of bone after 7 days was measured with a score (0 = to 3 = severe amount).

After 7 days of follow-up, connective tissue proliferation was the same at all temperatures tested, and no statistical group differences could be evaluated in this regard (Fig. 5A).

**Figure 6:**
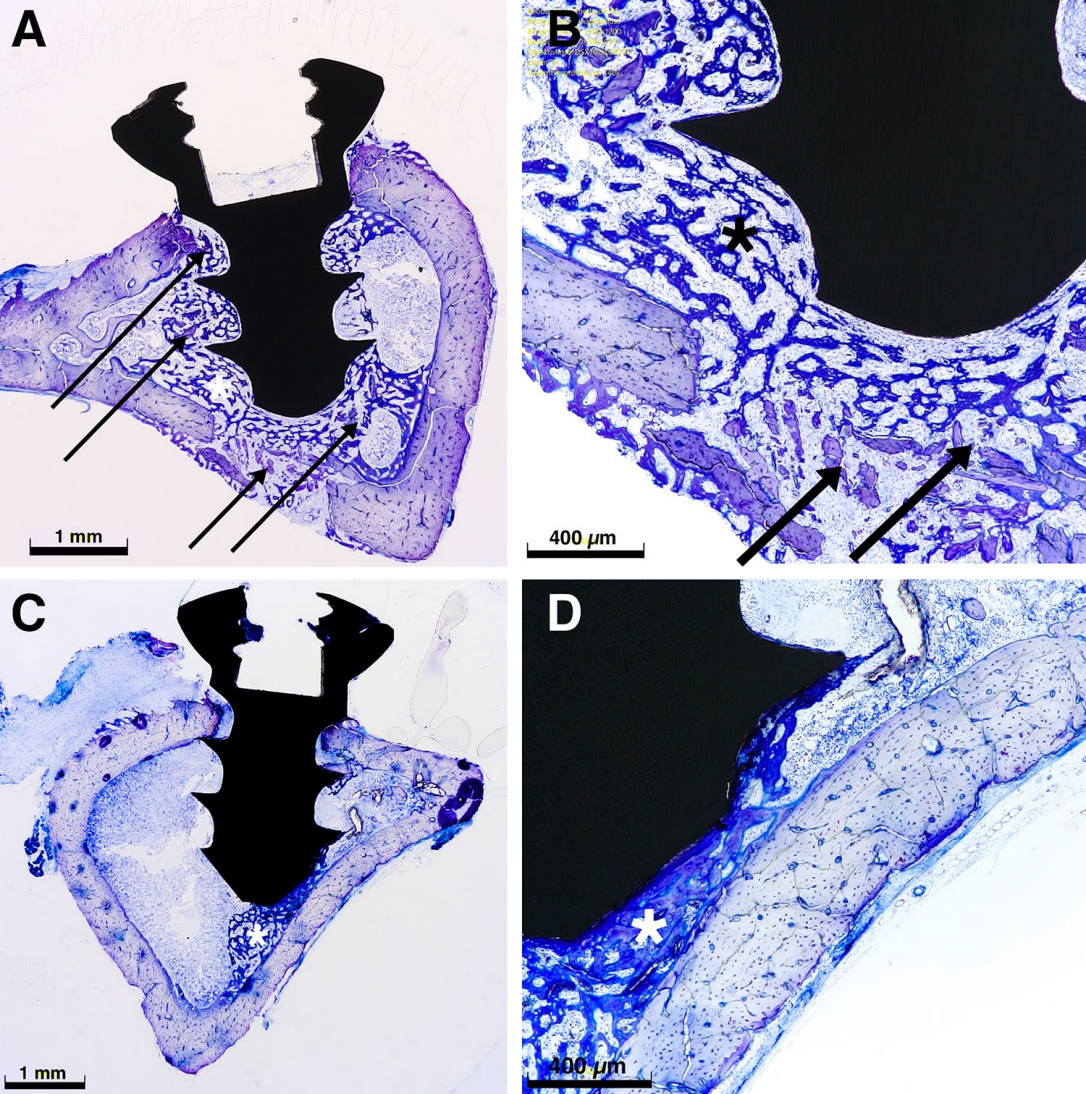
(**A**) Survey image of a test sample (50 °C, for a tempering time of 1 min, magnification of 42 ×) showing significant new bone formation adjacent to the implant (*) and necrosis at the implant shoulder (arrows), accentuated necrosis at the middle and the tip of the implant (arrows). (**B**) Close-up with a zoom of 140 ×, showing new bone formation (*) and evidence of necrosis (arrows) at the implant tip area. (**C**) Survey image of a control sample (no tempering, 42 ×) revealing mild to moderate new bone formation (*) predominantly adjacent to implant tip. No evidence of necrosis formation was measured. (**D**) Additionally, in the close-up of the control sample (140 ×), new bone formation without any evidence of necrosis was evaluated.

A similar result was recorded regarding the new bone formation; however, the value of 2 °C with no significance presented a lower value when compared with the remaining groups (Fig. 5B). In Fig. 6 tissue structures of light microscopy analysis are presented.

In Fig. 6A and B the image of a test sample (50 °C, for a tempering time of 1 min) is showing significant new bone formation adjacent to the implant and necrosis at the implant shoulder, accentuated necrosis at the middle and the tip of the implant. On the other hand, in Fig. 6B and C an image of a control sample (no tempering) revealing mild to moderate new bone formation is presented.

## Discussion

The goal of this rat study was to determine the temperature/time thresholds that would cause the least amount of damage to osteocytes and bony tissue, as the protocol's future in vivo implementation of thermo-explantation would necessitate that peri-implant bone necrosis is kept to a minimum. It is well known that exposing osteocytes to temperatures exceeding 45 °C for 15 s might cause injury^24^. Targeted cooling can produce an effect comparable to that of heating. Temperatures between 1 and 3.5 °C cause a histologically established effect on the bone and surrounding tissue (maximum 0.7 mm)^25,26^. According to a recent review, more preclinical research is needed based on these review findings to determine the influence of temperature and time intervals on the development of a restricted bone necrosis in the range of 47 °C to 55 °C for 1 min^21^. This animal study is based on a previously published preclinical pilot study that was carried out to investigate the potential of future explantation using thermally induced osteonecrosis^23^. The first cadaveric pilot study was conducted to reduce the sample sizes of this rat study, which was feasible, as temperature/time levels of 51 °C for 10 s and 5 °C for 30 s presented significant matrix degeneration. Based on these results, and considering the in vivo blood flow, temperature/time intervals of cold and warm temperatures 4 °C, 3 °C, 2 °C, 48 °C, 49 °C and 50 °C for a tempering time of 1 min were carried out.

Bone tissue is a diverse structure with cancellous and cortical areas that influence the temperature threshold of individuals^27^. Low-density bone has been found to be more susceptible to heat injury. Our results agree in this respect, since at 50° for 1 min more necrosis was evaluated in the area of the implant center at the tip compared with the cortical areal around the implant shoulder.

The BIC ratio can be used as an important parameter for reducing osseointegration, as it reflects the value of the bone contact area at the implant surface. The BIC ratio indicated decreasing values starting at warm temperatures of 48 °C. Above all, a significant difference was noted when comparing test group with 50 °C to the control group (p = 0.03). This agrees with the results of Trisi et al., who showed that at 50 °C for 1 min, BIC values significantly decreased and infrabony pockets increased^27,28^.

A possible explanation for the similar score results of new bone formation (Fig. 5B) may lie in the approach that both cold and warm temperatures can affect bone behavior. Normally, when bone formation is high, connective tissue formation is slow, and vice versa. Since our results do not reflect significance, no precise statement can be made about new bone formation versus connective tissue formation.

A critical reflection on the present study reveals that in this study design it is not the implants that were thermally treated but the drill holes. Due to the small size of the implant, it was technically not possible to reach the targeted temperature of 1 and 2 °C as the heat dissipation was too high and the cooling liquid froze. Furthermore, the implants were not osseointegrated before treatment. Therefore, defining reliable threshold temperature values requires in vivo investigations with osseointegrated implants. This rat study was conducted to reduce the sample sizes of subsequent animal studies in pigs with osseointegrated implants, as a successful thermo-explantation can only be validated on osseointegrated implants. Finally, this pig study will be feasible, as temperature/time level of 50 °C for 1 min presented significant BIC reduction.

## Conclusion

Although this is a preliminary in vivo study, the results identified heat temperatures and intervals to lower the number of samples in further studies of temperature-induced bone necrosis. Temperature/time level of 50 °C for 1 min presented a significant decrease of BIC ratio, at 49 °C already a trend development has been recorded. This level may be used for future thermo-explantation.

## Data Availability

The datasets used and/or analyzed during the current study available from the corresponding author on reasonable request.
